# Agenda setting and visit openings in primary care visits involving patients taking opioids for chronic pain

**DOI:** 10.1186/s12875-020-01317-4

**Published:** 2021-01-04

**Authors:** Eve Angeline Hood-Medland, Anne E. C. White, Richard L. Kravitz, Stephen G. Henry

**Affiliations:** 1 Department of Internal Medicine, University of California Davis, 4150 V Street Suite 2400, Sacramento, CA 95817 USA; 2 University of California Davis Center for Healthcare Policy and Research, Sacramento, CA USA

**Keywords:** Primary care, Chronic pain, Opioid analgesics, Physician-patient communication, Agenda setting, Conversation analysis, Mixed-methods

## Abstract

**Background:**

Agenda setting is associated with more efficient care and better patient experience. This study develops a taxonomy of visit opening styles to assess use of agenda and non-agenda setting visit openings and their effects on participant experience.

**Methods:**

This observational study analyzed 83 video recorded US primary care visits at a single academic medical center in California involving family medicine and internal medicine resident physicians (*n* = 49) and patients (*n* = 83) with chronic pain on opioids. Using conversation analysis, we developed a coding scheme that assessed the presence of agenda setting, distinct visit opening styles, and the number of total topics, major topics, surprise patient topics, and returns to prior topics discussed. Exploratory quantitative analyses were conducted to assess the relationship of agenda setting and visit opening styles with post-visit measures of both patient experience and physician perception of visit difficulty.

**Results:**

We identified 2 visit opening styles representing agenda setting (agenda eliciting, agenda reframing) and 3 non-agenda setting opening styles (open-ended question, patient launch, physician launch). Agenda setting was only performed in 11% of visits and was associated with fewer surprise patient topics than visits without agenda setting (mean (SD) 2.67 (1.66) versus 4.28 (3.23), *p* = 0.03).

**Conclusions:**

In this study of patients with chronic pain, resident physicians rarely performed agenda setting, whether defined in terms of “agenda eliciting” or “agenda re-framing.” Agenda setting was associated with fewer surprise topics. Understanding the communication context and outcomes of agenda setting may inform better use of this communication tool in primary care  practice.

## Background

Agenda setting is a communication strategy physicians use at the beginning of clinical visits to elicit, propose, and organize a complete list of topics to be covered [1]. Topics are clinical issues raised by either patient or physician [2]. Agenda setting is thought to improve patient outcomes and experience [3], physicians’ understanding of patients’ concerns [4], and physician organization and time management by reducing the number of unaddressed concerns and, by extension, the number of “surprise” topics patients introduce later in the visit [5, 6]. Agenda setting is a standard skill taught to medical students and residents, does not significantly affect visit length [7–9], and is accepted as best practice [10–12]. Physicians, however, rarely perform agenda setting [13, 14], which can result in more frequent unaddressed concerns [4, 9, 15, 16]. Studies have shown that training physicians in agenda setting and visit organization strategies can result in improved communication, particularly by reducing the introduction of surprise topics [9, 17].

There are several existing gaps in research on agenda setting. Previous research found a large variation of its occurrence, ranging from 32 to 68% of visits [4, 9, 13, 14, 18]. A lack of a standard agenda setting definition across studies is likely an important but underappreciated cause of this variability. For instance, some studies include all visits that start with an open-ended question in their agenda setting definition [14] while others have set time limits (e.g., the first five minutes [18]) for when agenda setting must occur.

Previous studies of agenda setting have predominantly focused on visits addressing new patient concerns. Relatively little is known about physician behavior and visit organization in follow-up visits for chronic conditions, which comprise the majority of primary care visits [19, 20]. Visits for chronic pain are an example of challenging chronic care conditions that are worthy of attention due to their prevalence, impact on quality of life, and their influence on physician perception of visit difficulty [21–23]. Additionally, chronic pain can take a substantial amount of visit time during which multiple other chronic problems must also be addressed [24, 25]. Patients themselves can bring multiple concerns to a single visit [8, 26–29], and physicians must also address many guideline-based clinical directives.

Limited data exists for the specific impact of agenda setting on patient experience and physician perception of visit difficulty [22, 23]. Taking the physician perspective into consideration is important given the current prevalence of physician dissatisfaction and burnout, which in turn can decrease patient centeredness and increase physician turnover [30–35]. Physician-reported visit difficulty has also been associated with worse patient experience and higher healthcare utilization [36, 37]. Communication strategies are needed to assist physicians in navigating “difficult” visits [23], and this study specifically examines visits focused on chronic pain and opioids, which have been associated in other studies with high levels of physician-reported difficulty [23, 38].

By examining chronic care visits, our study sought to address these knowledge gaps by pursuing the following goals: 1) characterize primary care physicians opening styles within the framework of agenda setting as a first step towards developing a standardized definition of agenda setting 2) assess associations between agenda setting and a) topics discussed (e.g., surprise topics), b) patient experience ratings, and c) physicians’ perception of visit difficulty. Chronic pain is an example of a symptom-driven chronic condition that is broadly representative of other chronic conditions seen in primary care [39]. This study expands on current knowledge on agenda setting and is important because observations from patient-physician interactions can help inform next steps in educational and health system priorities around organizing chronic care visits and communicating about chronic conditions such as chronic pain.

## Methods

This is a qualitative, observational study. We first used conversation analysis [40, 41] to create a taxonomy of visit opening styles. We then applied this inductively derived taxonomy to our data and examined quantitative associations between these categories and topics discussed and post-visit measures of patient experience and physician perception of visit difficulty.

### Data sources and participants

Data sources were 86 video recorded clinical visits and associated patient and resident physician questionnaires. Three of the 86 recorded encounters were excluded from our study because they did not include the initial opening sequence, leaving 83 encounters in our study. Physicians were second- or third-year internal medicine or family medicine residents at the University of California Davis Medical Center. Patients were established adult patients planning to discuss pain management with an enrolled physician during a routine appointment. Patients were ineligible if they spoke a language other than English during visits, were getting active cancer treatment or palliative care, or were receiving an opioid prescription from someone other than their primary care physician. Patient and physician demographic information were collected at enrollment. The University of California Davis Institutional Review Board approved the study. Written consent was obtained from all participants, and detailed study procedures have been previously described [42, 43].

### Participant experience measures

After each visit, physicians completed the 10-item Difficult Physician-Patient Relationship Questionnaire [44]. Physician difficulty scores could range from 10 to 60 and higher scores represent more difficult visits. Patients completed 4 measures of patient experience: the short form of the Wake Forest trust scale [45], a 3-item measure of agreement with treatment plan [46], an assessment of physician communication skills from the Consumer Assessment of Healthcare Providers and Systems (CAHPS) Adult Visit Survey [47], and a patient-facing version of the Difficult Physician-Patient Relationship Questionnaire described in prior studies by Henry et al. [43] Exploratory factor analysis indicated that all 4 measures assessed a single latent construct; therefore, these 4 measures were combined into a single standardized (population mean = 0, SD = 1) measure of patient experience, with higher values indicating a better experience [43].

### Coding procedures

Two authors, a primary care physician and a medical sociologist trained in conversation analysis [40, 41] (AECW) watched study video recordings and coded visits together. We worked in tandem to determine two coding schemes for *Visit Openings* and for *Topic-Level* review*.* Our complementary expertise allowed us to simultaneously analyze data both for medical *content* and for interactional *process*, allowing for richer analysis. For instance, a conversation analyst may not recognize when one medical topic shifts into another topic. A physician may not recognize the communicative practices speakers use to accomplish shifting between topics.

### Qualitative analysis: visit openings

First, we determined the *visit opening* style by watching visits from their onset through the first topic discussed. Initially, we anticipated developing a binary coding scheme indicating whether agenda setting occurred or not. After reviewing 20 videos, however, we realized we needed a more nuanced coding of agenda setting, which led to an inductively-driven analysis of visit openings (still based on the basic purpose of generating an upfront list of topics) informed by conversation analysis principles which focus on how participants begin an interaction [16, 48], introduce topics for discussion [29, 49], transition from one topic to the next [50], and analyzes the overall sequential order in which topics are discussed [51].

We defined agenda setting as strategies to explicitly elicit or confirm an upfront list of agenda topics *before* discussing the first topic, and our definition did not include an arbitrary time limit. Previous research has shown that broad open-ended questions during visit openings (e.g., “What can I do for you today?”) typically generate a single topic and are ineffective in soliciting an upfront list of topics [13, 16], so open-ended questioning by itself did not fulfill our definition. We expanded our analysis to include the opening sequence of 45 visits, at which point we reached saturation. In tandem we then applied the final visit opening coding scheme to all 83 visits. Disagreements were negotiated and a conclusion achieved by consensus.

### Qualitative analysis: topic-level

Subsequently, we performed in tandem a *topic-level* review of 15 visits, at which point we reached saturation for development of a coding schema for topic-related variables. This entailed watching visits from when physicians entered the room until they exited. This initial review established a coding scheme for the types of topics discussed and how to represent their occurrence. This inductively-driven analysis led to the final list of topic-related variables. We coded for the frequency of the following: total topics, major topics, surprise topics, return topics, and the length of the visit (see Table 3 for definitions). We coded topics initiated by patient companions as patient-initiated topics. Disagreements were negotiated and a conclusion achieved by consensus.

We then applied this final topic-level coding scheme to a subset of 41 videos due to the time intensive nature of tandem coding (each visit took approximately 2 h to code) and coordinating research schedules. We purposefully selected all visits with agenda setting for topic-level coding (*n* = 9), and we selected 32 additional visits using maximum variation sampling to represent family medicine and internal medicine resident physicians, a proportionate distribution of the visit opening styles, and a wide range of patient experience and physician difficulty scores [53].

### Quantitative analysis: visit openings

Using the whole sample (*n* = 83), we constructed separate linear regression models with patient experience and physician perception of visit difficulty as dependent variables and agenda setting (present/absent) as the independent variable. We then ran 2 additional regressions with the same 2 dependent variables analyzing visit opening style as 5-level categorical variable. Open-ended question visit opening style was the reference group for all analyses using the 5-level categorical variable.

### Quantitative analysis: topic-level

Using the 41 visits that underwent topic-level coding, we constructed separate linear regression models to assess for differences in means of 5 dependent variables (total topics, major topics, surprise topics, return topics, length of visit) among groups defined by agenda setting (present/absent) as the independent variable. We then ran additional regressions with the same variables analyzing visit opening style as a 5-level categorical variable.

All analyses controlled for standard demographics (patient age, sex, and white versus nonwhite race) that may act as confounders, and used general estimating equations to account for clustering of patients within physicians. Analyses were conducted using SAS 9.4.

## Results

The 83 visits coded for visit opening style had a mean patient experience standardized score of 0.02 (SD = 0.87) and a mean physician difficulty score of 27.4 (SD = 10.71). Table 1 provides demographic information for patients and physicians.

**Table 1:** Patient and Physician Demographics

	Patients *n* = 83	Physicians *n* = 49
Age		
< 30	0	31
30–39	5	17
40–49	7	1
50–64	42	0
65+	29	0
Sex		
Male	30	12
Female	53	37
Race		
White	56	23
Non-White	27	26

### Qualitative results: conversation analysis of visit openings

Visits demonstrated 5 distinct visit opening styles (see Table 2 for definitions and example transcripts). We found 3 opening styles that did not qualify as agenda setting: *open-ended question*, *patient launch*, and *physician launch*. The non-agenda setting openings launched into a first topic without establishing, at least on a *pro tem* basis, the full set of topics to be discussed.

**Table 2:** Descriptions and examples of visit openings

Type of Visit Opening	Definition	Example visits (target lines that define the visit opening have been bolded)
**NO AGENDA SETTING**	**Physician and patient discuss topic(s) without physician first agenda setting.**	See examples in open-ended question, patient launch, and physician launch.
Open-ended question	Physician begins visit by asking a broad open-ended question. In response, the patient proposes a first topic and this topic then gets discussed. This open-ended question does not explicitly ask for list of topics, nor is it followed by agenda reframing.	01 PAT: Hello Dr. <Name>. 02 **DOC: What brings you in today?** 03 PAT: I was trying to get a paper to bring in for you to fill it out, but I didn’t get it. 04 DOC: Paper for what? 05 PAT: Uh- 06 DOC: Disability? 07 PAT: No, from ((inaudible)) housing. 08 DOC: Oh, okay. 09 PAT: You know how they say you’re only eligible for one bedroom apartment? 10 DOC: Mm hmm 11 PAT: And the lady -- uh -- has said -- I was telling her, I said, “Well, I stayed in 12 ((location)),” I said, but they told me I couldn’t stay there by myself. 13 DOC: Mm hmm 14 PAT: I said, so I’m not there anymore. So. She say she mailed tomorrow and get it. 15 DOC: Where are you living now? 16 ((patient’s housing situation continues to get discussed)) Pt 118
Patient launch	Patient begins visit by initiating a first topic, and physician pursues this topic.	01 **PAT: Okay. The first thing I wanna ask is the hospital called me about- for pain** 02 **management from the spine clinic**. 03 DOC: Mm hmm 04 PAT: Um, but they won’t do anything until after you give the okay. 05 DOC: Okay. So, my question is, um, I’m not sure if I should- who -did they say 06 exactly how I was supposed to give the okay? Pt 314
Physician launch	Physician begins visit by initiating and pursuing a first topic.	01 **DOC: So, the last time you came to our clinic, you had a cough. How is that** 02 **doing?** 03 PAT: Uhh, still around, right? But it’s kind of leaving. Like, for one, she didn’t give me 04 enough medication. 05 DOC: Prednisone, or-? Pt 249
**AGENDA SETTING**	**Physician sets an agenda before discussing first topic.**	See examples in agenda eliciting and agenda reframing.
Agenda eliciting	Physician begins visit by explicitly asking patient for a list of their topics. While this question is open-ended, the inquiry solicits a narrowed topic list [52].	**((visit opens with greetings; COM = patient’s companion))** 07 **DOC: Was there anything in particular you guys wanted to address?** 08 COM: His potassium level 09 DOC: Yeah. Okay. 10 COM: His phantom pain. 11 DOC: Uh huh 12 COM: And uh, the chest X-ray. We never really discussed that last time. Pt 17
Agenda reframing	Visit begins by either the physician asking a broad open-ended question or the patient launching into a first topic (see definitions below). The physician, however, does *not* engage with the patient’s proposed first topic but instead reframes the patient’s talk into a visit agenda. This definition requires that the opening lines do not meet the definition of agenda eliciting; agenda reframing is counted as agenda setting because the physician pauses to establish the agenda before discussing the first clinical topic.	01 DOC: What can I do for you today? 02 PAT: I’m not doing no good. 03 DOC: Oh, not doing so good? Why is that? 04 PAT: ‘Cuz I’ve been having fever now and then. Then I started coughing, and by now, 05 I’m coughing a lot. 06 DOC: Okay. 07 PAT: And yesterday, I couldn’t even talk, my throat was so bad. 08 DOC: Oh, was it a sore throat? 09 PAT: Mm hmm 10 **DOC: Okay. You were coming in today to talk about fever, cough, all those things?** 11 PAT: Yeah, ‘cuz I called over here because I haven’t checked my Coumadin yet. 12 DOC: Mm hmm 13 PAT: She told me since I was gonna come over here, it was worth it for me to come and 14 see the doctor. 15 DOC: Okay. 16 PAT: ‘Cuz my legs feel real restless. 17 **DOC: Okay. So, well, let’s—we’ve got to, you know, decide, you know, a couple of** 18 **things to talk about today. It sounds like number one, you were coming in-** 19 **you have fever and a cough, sore throat?** 20 PAT: Mm hmm 21 **DOC: Then you say that another thing is, your legs feel restless**. 22 PAT: Mm hmm. 23 DOC: Okay. 24 PAT: My legs feel restless a lot. I can’t even stand it sometimes. 25 DOC: Okay. 26 PAT: I keep rubbing my legs on the bed, or one or the other, and it still won’t go away. 27 **DOC: Okay. All right. Let’s talk about, first, the fever and cough and all that** 28 **stuff. When did that start?** Pt 432

We found two distinct visit opening styles physicians used to perform agenda setting. The first style was *agenda eliciting*, which is a standard approach taught to medical students and residents. In this approach, physicians request from patients an upfront list of their medical concerns (e.g.,“What are the main things we want to talk about?”). The second style we identified was *agenda reframing*, as it allows physicians to reformulate the patient’s talk at the beginning of the visit (which could be about one or more potential topics to be discussed) into an explicit agenda.

Using conversation analysis, we demonstrate a case of agenda reframing to provide a detailed description of this novel conceptualization of agenda setting (see Table 2 for transcript). The visit begins with the physician asking an open-ended question, “What can I do for you today?”, a standard agenda eliciting opening. However, instead of conforming to the topic of the question, the patient responds to this general inquiry as if it were a “How are you” question [16] with, “I’m not doing no good.”, which the physician unpacks in line 3. The patient then begins to describe the array of concerns she is suffering from including fever, coughing, and a sore throat. Instead of launching into an investigation of these concerns, the physician tries to reframe these concerns as a *list* of topics (line 10).

In response to the physician’s first attempt to have her agree to an agenda, the patient provides only a token confirmation, “Yeah” (line 11), and she then rushes into her next-turn-at-talk (with a compressed “cuz”) about another topic—a question about a prescription and its potential relatedness to having restless legs. At this point, the physician shifts the conversation away from the patient’s attempted launch into the restless leg topic, and again tries to synthesize the patient’s concerns into an upfront agenda while also negotiating what the priorities of the visit are and in what order these topics should be discussed (lines 17–19, 21). While the physician is attempting to get the patient to recognize the act *and* content of agenda setting, the patient does neither. The patient transforms the physician’s confirmation question about restless legs (and the topic being on the agenda (line 21)) into a request for more information and as a launch into the topic of her legs. This is evident in the patient’s elaboration about her legs (lines 24, 26).

For the third and final time, the physician repeatedly refrains from following the patient’s attempted path into a discussion about a medical topic before establishing an agenda, and again pauses to set the agenda. The physician now does so with a declarative formulation of the agenda to “first” discuss the fever and the cough (line 27), which implicitly leaves the restless leg topic as the subsequent topic. Only then, after having established an agenda unilaterally after two failed collaborative attempts, does the physician move out of the opening phase of the visit and into the history taking phase (line 28).

While this excerpt may show an exceptional amount of demonstrated restraint by the physician to curtail the patient’s many attempts to delve straightaway into a medical topic, this physician has successfully shown how agenda reframing potentially helps make the visit less disorganized than it would have been otherwise. Agenda reframing is a helpful practice when patients, as demonstrated here, do not readily provide an upfront list of topics to an agenda eliciting question (or to other open-ended question visit openings). Physicians can also use agenda reframing when patients begin a visit with a patient launch. Agenda reframing allows physicians to hit the brakes while still incorporating the concerns raised by the patient into the potential agenda.

### Qualitative results: topic-level

Table 3 defines the topic-level variables we assessed for each visit (total topics, major topics, surprise topics, return topics) and provides an illustrative example visit.

**Table 3:** Topic-level review definitions with an illustrative example

Patient #78 Visit This example visit shows who initiated each topic and the chronological order in which topics were discussed. This visit had a patient launch visit opening 9 (defined in Table 2), as evident by video review and not by looking at this topic list.	Time 0.00 PAT: Falling DOC: Stomach issue PAT: Psychosocial PAT: Nerve pain PAT: Stomach issue PAT: Falling PAT: Chronic pain PAT: Care management DOC: Chronic pain PAT: Falling DOC: Cholesterol DOC: Smoking cessation PAT: Care management DOC: Smoking cessation PAT: Chronic pain PAT: Psychosocial DOC: Chronic pain Doctor leaves room 18:22	
**Variable**	**Definition**	**Value in example above**
Total topics	Count of unique topics discussed.	*n* = 8 falling, stomach issue, psycho-social, nerve pain, chronic pain, care management, cholesterol, smoking cessation
Major topics	Count of topics that received a comprehensive discussion. Determined by physician coder (EAMH) after reviewing full visit. Determined by video review and not by looking at topic list.	*n* = 1 chronic pain
Surprise topics	Count of total topics patients brought up that were not agenda items. If no agenda setting occurred, all patient-initiated topics were considered surprise topics for the physician.	*n* = 6 falling, psychosocial, nerve pain, stomach issue, chronic pain, care management
Return topics	Count of topics mentioned more than once. A single topic that was returned to more than once was counted as multiple return topics.	*n* = 9 falling 2x, stomach issue 1x, psychosocial 1x, chronic pain 3x, care management 1x, smoking cessation 1x

This visit begins with the patient launching into a first topic about his recent fall off a moving truck, and the visit proceeds without the physician pausing to set an agenda. Because the physician does not solicit an upfront list of topics from the patient, every patient-initiated topic throughout the visit is therefore an unanticipated surprise topic for the physician. All but 2 topics (stomach issues and smoking cessation) are patient-initiated surprise topics. This topic-level review allowed us to ascertain not only the types of topics discussed but also how often the same topic gets returned to (e.g., chronic pain gets returned to 3 times). While this particular visit only has one major topic (chronic pain) that receives a comprehensive discussion, there are 8 total topics discussed.

### Quantitative results: visit openings

We found the 3 opening styles that did not qualify as agenda setting comprised the vast majority of the visits: *open-ended question* (*n* = 41), *patient launch* (*n* = 15) and *physician launch* (*n* = 18), while the 2 opening styles that qualified as agenda setting occurred relatively infrequently: *agenda eliciting* (*n* = 6) and *agenda reframing* (*n* = 3). In total, 9 of the 83 visits (11%) included agenda setting.

We re-categorized these 5 visit opening styles into a 2-level variable of those visits that met the agenda setting definition (agenda eliciting, agenda reframing) and those that did not (open-ended question, patient launch, physician launch). There was no statistically significant difference in patient experience and physician perception of visit difficulty for visits in which agenda setting was present versus absent, (Table 4) nor among the 5 visit opening styles.

**Table 4:** Mean patient experience and physician difficulty with or without agenda setting

All Visits^a^ *n* = 83	Agenda Setting *n* = 9	No Agenda Setting *n* = 74		
	Mean (SD)	Mean (SD)	Beta (95%CI)	*p*-value
Patient experience^b^	−0.60 (1.60)	0.09 (0.72)	−0.70 (− 1.70, 0.3)	0.18
Physician perceived difficulty^c^	31.11 (10.43)	26.89 (10.72)	4.20 (−2.80, 11.30)	0.24

^a^Visits are the 83 that were reviewed for visit opening style

^b^Patient experience: Single standardized measure of four measures (short form of the Wake Forest trust scale [45], 3-item measure of agreement with treatment plan [46], Consumer Assessment of Healthcare Providers and Systems (CAHPS) Adult Visit Survey [47], patient-facing version of the Difficult Physician-Patient Relationship Questionnaire) [43] with higher values indicating a better experience

^c^Physician perceived difficulty: 10-item Difficult Physician-Patient Relationship Questionnaire, scaled 0–60 with 60 being most difficult [44]

*SD* Standard Deviation, *CI* Confidence Intervals

### Quantitative results: topic-level

The 41 visits coded for topics discussed had a mean length of 25.6 min (SD 7.12), and a mean of 8.10 (SD = 3.47) total topics discussed. Visits averaged 1.63 (SD = 0.70) major topics and 7.85 (SD = 4.14) returns to prior topics. Visits averaged 3.93 (SD = 3.01) surprise topics. All visits had at least 1 surprise topic.

We found visits with agenda setting had a statistically significantly lower number of surprise topics (mean = 2.67, SD = 1.66) compared to visits without agenda setting (mean = 4.28, SD = 3.23) (*p*-value = 0.03) (Table 5). No significant differences were found in visit length, number of total topics covered, return topics, or number of major topics discussed between visits with or without agenda setting. No significant differences were found for any dependent variables among the 5 different visit opening styles.

**Table 5:** Visit topic variables with or without agenda setting

Topic Level^a^ *n* = 41	Agenda Setting *n* = 9	No Agenda Setting *n* = 32		
	Mean (SD)	Mean (SD)	Beta (95%CI)	*p*-value
Total topics	7.33 (2.35)	8.31 (8.73)	−0.70 (−2.30, 0.89)	0.38
Major topics	1.78 (0.44)	1.59 (0.76)	0.23 (−0.15, 0.61)	0.23
Surprise topics	2.67 (1.64)	4.28 (3.22)	−1.38 (−2.57, −0.20)	0.03
Return topics	7.11 (3.72)	8.06 (4.29)	−1.05 (−3.86, 1.75)	0.46

^a^Visits are the 41 that were reviewed for visit opening style

*SD* Standard Deviation, *CI* Confidence Intervals

## Discussion

In this study examining physicians’ agenda setting in primary care visits for patients taking opioids for chronic pain, we developed a taxonomy of visit opening styles. We identified two distinct methods physicians used to set an agenda: agenda eliciting and agenda reframing. This study is the first to identify and describe *agenda reframing*, which is a practice physicians use to reformulate the patient’s talk at the beginning of the visit (which could be about one or more potential topics to be discussed) into an explicit agenda. Our study also confirmed the importance of agenda setting, as our exploratory quantitative analysis found that any use of agenda setting was associated with fewer surprise topics, but no form of visit opening style was associated with a change in patient experience or physician perception of visit difficulty.

We found that resident physicians performed agenda setting in only 11% of chronic care visits. Almost 50% of visits started with a broad open-ended question that then transitioned into the first topic without physicians pausing to establish (or reframe) the agenda. Thus, open-ended questioning does not, by itself, reliably establish a complete visit agenda. This finding suggests open-ended questioning should not be included in the definition of agenda setting. Even though our agenda setting frequency is lower than other studies of recorded visits [4, 9, 13, 14, 18], a finding potentially attributable to our relatively constrained agenda setting definition, we believe our definition is a more accurate representation of the phenomena and will set a more clear rubric for future studies.

Our low rate of observed agenda setting may also be related to physician preference, perceived lack of time, lack of comfort with agenda setting, lack of education about agenda reframing as a method, or physicians taking a tailored approach to particular patients. Furthermore, patient behavior may also curtail physicians’ best efforts to agenda set and may reflect unique challenges in a chronic care environment, where patients and physicians negotiate multiple topics. These results resonate with the work of Stuart et al. [54] in the UK, who found that physicians often delay soliciting additional concerns until the end of the visit. Future studies should assess which patient- or physician-related factors influence agenda setting. Understanding these influences could inform pre-visit interventions, potentially leading to better visit experiences.

We next address studying agenda setting in the context of chronic pain. Despite indications that chronic pain can dominate visits and distract attention from other clinical issues [55–58], our data show participants addressed a multitude of topics (an average of 8 per visit). Our finding exceeds the number of total topics discussed in Brock et al.’s study which compared visits with and without agenda setting (an average of 4.75 and 5.15 per visit, respectively) [7]. One potential explanation is that patients in resident clinics tend to have more complicated chronic health concerns and transportation issues that may encourage physicians to address more topics [59, 60]. Furthermore, recent studies found patients who take opioids for chronic pain receive improved care because more frequent visits provide opportunities for more preventive care topics to be addressed [61, 62].

In our analysis of surprise topics, we found that agenda setting was associated with fewer surprise topics, which could be attributed to the inherent benefit of agenda setting—having physicians elicit an “unsurprising” upfront list of topics at the beginning of the visit. This finding aligns with other studies [7, 13], suggesting our definition of agenda setting, which excludes open-ended questions if performed without agenda reframing, has some construct validity. Averting surprise topics could improve quality of care by shifting critical discussions earlier in the visit, where they are likely to be afforded more time [2]. An important caveat is that all 9 of the agenda setting visits ultimately contained at least one surprise topic. Since surprise topics can occur despite agenda setting, physicians may view agenda setting as ineffective.

We did not find differences in physician perception of visit difficulty between visits with or without agenda setting. We theorized that if physicians generally perceive visits for chronic pain as difficult [22], there may not be sufficient variation in visits to detect a change. We did not have a comparison group of non-pain visits. Future studies could investigate more diverse chronic care visits. Physician wellbeing is part of the quadruple aim which expands on the triple aim of improving healthcare (through better outcomes, lower costs, improved patient experiences) by including staff wellbeing [63, 64]. Developing improved communication skills for use in this patient population could improve the patient-physician experiences [36, 65].

The strengths of this study include direct observation of clinical visits using videotape and use of mixed analytic methods, but like all studies ours has limitations. This study took place at two clinics in a single academic health center, limiting generalizability to other settings. However, findings from this setting are highly relevant to medical education. Additionally, our sample size limited our statistical power to identify small differences between visits with and without agenda setting. Our study measured the number of topics discussed per visit but did not examine how effectively topics were discussed.

Along with prior studies, our work demonstrates that agenda setting may be a useful strategy for reducing surprise topics. A unique finding from our study is identifying the practice of agenda reframing, which has not to our knowledge been formally taught but seems to be a viable agenda setting strategy. Agenda reframing can be potentially taught as a simple 3 step process: 1) *Ask ‘*What brings you in today?’ 2) *Encourage* patients to expound 3) *Redirect* to additional items on the agenda. Teaching physicians multiple strategies for agenda setting (i.e. agenda eliciting or agenda reframing) may help physicians feel more comfortable setting agendas. Of course, further research is needed to explore these hypotheses. Because agenda setting is a free communicative intervention, does not make visits longer, and can provide benefits such as reducing surprise topics, we believe our findings match current consensus that agenda setting is a valuable strategy.

## Conclusion

In this study examining physicians’ agenda setting in primary care visits for patients taking opioids for chronic pain, we developed a taxonomy of visit opening styles which comprised 2 styles of agenda setting (*agenda eliciting* and *agenda reframing*) and 3 styles of non-agenda setting (*open-ended question*, *patient launch, or physician launch*). Resident physicians rarely perform agenda setting with patients who have chronic pain. When performed, it was associated with fewer surprise patient topics, but no form of visit opening style was associated with a change in patient experience or physician perception of visit difficulty. Understanding the use of agenda setting in visits for chronic problems may help primary care physicians to decide the best use of this communication tool in their practice.

## Data Availability

Some data in this study are confidential. The video recordings contain identifiable data and so are not publicly available. But the datasets generated and analyzed during the study are available subject to required ethical and regulatory approvals. Those interested in using these data should contact the senior author (SGH).
